# Diversity of *KIR* genes and their *HLA-C* ligands in Ugandan populations with historically varied malaria transmission intensity

**DOI:** 10.1186/s12936-021-03652-y

**Published:** 2021-02-25

**Authors:** Stephen Tukwasibwe, James A. Traherne, Olympe Chazara, Jyothi Jayaraman, John Trowsdale, Ashley Moffett, Wei Jiang, Joaniter I. Nankabirwa, John Rek, Emmanuel Arinaitwe, Samuel L. Nsobya, Maxine Atuheirwe, Mubiru Frank, Anguzu Godwin, Prasanna Jagannathan, Stephen Cose, Moses R. Kamya, Grant Dorsey, Philip J. Rosenthal, Francesco Colucci, Annettee Nakimuli

**Affiliations:** 1 Department of Obstetrics and Gynaecology, School of Medicine, Makerere University College of Health Sciences, P.O BOX 7072, Kampala, Uganda; 2 Infectious Diseases Research Collaboration, 2C Nakasero Hill Road, Kampala, Uganda; 3 Department of Pathology, University of Cambridge, Cambridge, UK; 4 University of Cambridge Centre for Trophoblast Research, Cambridge, UK; 5 Stanford University, School of Medicine, Stanford, USA; 6 MRC/UVRI and LSHTM Uganda Research Unit, Kampala, Uganda; 7 University of California, San Francisco, USA; 8 Department of Obstetrics & Gynaecology, University of Cambridge, National Institute for Health Research Cambridge Biomedical Research Centre, Cambridge, CB2 0SW UK

**Keywords:** Genetic diversity, Human leukocyte antigen, Killer-cell immunoglobulin-like receptor, Malaria, Uganda

## Abstract

**Background:**

Malaria is one of the most serious infectious diseases in the world. The malaria burden is greatly affected by human immunity, and immune responses vary between populations. Genetic diversity in *KIR* and *HLA-C* genes, which are important in immunity to infectious diseases, is likely to play a role in this heterogeneity. Several studies have shown that *KIR* and *HLA-C* genes influence the immune response to viral infections, but few studies have examined the role of *KIR* and *HLA-C* in malaria infection, and these have used low-resolution genotyping. The aim of this study was to determine whether genetic variation in *KIR* and their *HLA-C* ligands differ in Ugandan populations with historically varied malaria transmission intensity using more comprehensive genotyping approaches.

**Methods:**

High throughput multiplex quantitative real-time PCR method was used to genotype *KIR* genetic variants and copy number variation and a high-throughput real-time PCR method was developed to genotype *HLA-C1* and *C2* allotypes for 1344 participants, aged 6 months to 10 years, enrolled from Ugandan populations with historically high (Tororo District), medium (Jinja District) and low (Kanungu District) malaria transmission intensity.

**Results:**

The prevalence of *KIR3DS1, KIR2DL5, KIR2DS5*, and *KIR2DS1* genes was significantly lower in populations from Kanungu compared to Tororo (7.6 vs 13.2%: p = 0.006, 57.2 vs 66.4%: p = 0.005, 33.2 vs 46.6%: p < 0.001, and 19.7 vs 26.7%: p = 0.014, respectively) or Jinja (7.6 vs 18.1%: p < 0.001, 57.2 vs 63.8%: p = 0.048, 33.2 vs 43.5%: p = 0.002, and 19.7 *vs* 30.4%: p < 0.001, respectively). The prevalence of homozygous *HLA-C2* was significantly higher in populations from Kanungu (31.6%) compared to Jinja (21.4%), p = 0.043, with no significant difference between Kanungu and Tororo (26.7%), p = 0.296.

**Conclusions:**

The *KIR3DS1*, *KIR2DL5*, *KIR2DS5* and *KIR2DS1* genes may partly explain differences in transmission intensity of malaria since these genes have been positively selected for in places with historically high malaria transmission intensity. The high-throughput, multiplex, real-time *HLA-C* genotyping PCR method developed will be useful in disease-association studies involving large cohorts.

## Background

Malaria is estimated to cause nearly half a million deaths each year worldwide [1]. Malaria is a known evolutionary driving force in the selection of several human genetic polymorphisms that protect against malaria. Red blood cell alterations are the most studied genetic abnormalities that impact on malaria [2]. These include mutations in the alpha- and beta-globin genes that lead to sickle cell anaemia or thalassemias, glucose-6-phosphate dehydrogenase (G6PD) deficiency and the Duffy antigen protein [3]. It has been suggested that many of these polymorphisms were selected in human populations due to their role in protection from the detrimental effects of *Plasmodium falciparum* infection [4]. It has been demonstrated that different populations have developed independent evolutionary responses to malaria [5]. For example, 3 haemoglobin variants (HbS, HbC, and HbE) appear to confer protection against malaria in different parts of the world [6]. The HbS allele is common in Africa, but rare in Southeast Asia, and the opposite is true for the HbE allele [7, 8].

A recent genome wide association study of 17,000 individuals in Africa reported that known genetic variants account for only 11% of the total genetic influence of malaria on the human genome [9]. Among other genes potentially influencing malaria responses are those mediating innate immunity, which is important in protection from *P. falciparum* infection. Natural killer (NK) cells play an important role in the innate immune response to malaria infection [10, 11]. NK cells are the first cells in peripheral blood to produce interferon gamma (IFN- γ) in response to *P. falciparum* infection [11], and they have also been shown to participate in adaptive immunity. Recent evidence indicates a role for NK cells in malaria infection in humans and in mouse models [10, 12]. It has been shown that copy number variation (CNV) in *KIR* genes influences immunity to infections [13] and plays an important role in NK cell education [14] through interactions with their *HLA* class I ligands. Hence, the expression of multiple copies of *KIR* genes could potentially lead to enhanced NK cell education, thereby strengthening immunity to pathogens. This has been well studied in viral infections, but not in malaria.

Some studies have demonstrated that individuals may vary in their ability to elicit an innate immune response to malaria infection, with clear implications for disease manifestations [15]. Heterogeneity in response could arise from variations in KIR and their major ligands, HLA-C molecules, that have a direct impact on NK cell functions [11, 16]. The frequencies of different *KIR* and *HLA-C* genes vary remarkably across world populations, which might reflect differential selection pressures as well as persistence of ancestral genotypes [17]. The *KIR* and *HLA* loci have been suggested to be fast evolving and under positive selection, with pathogen pressure as the driving force [18, 19]. Genetic variation of *KIR* and their *HLA-C* ligands across the African continent is not well documented. Several studies have linked high *KIR* and *HLA* genetic diversity in Africa to malaria pressure [20–22]. However, there is limited data regarding the distribution of *KIR* and *HLA* variants in populations with varied malaria transmission intensity. Since interactions between the genetically diverse KIR and HLA molecules modulate the functionality of the NK cell response to malaria infections, a better understanding of the distribution of *KIR* and *HLA* genes in populations with varied malaria transmission intensity will be important in appreciating the impact of *P. falciparum* malaria on the evolution of *KIR* and *HLA* genes.

To date, there is limited data on the distribution of *KIR* and their *HLA-C* ligands in populations with varied malaria transmission intensity. This is partly due to the genotyping approaches that only reveal information about presence or absence of *KIR* and *HLA* genes. Furthermore, the few studies that have been carried out are case–control comparisons of severe *versus* uncomplicated malaria, with limited genetic information about *KIR* and *HLA* genes. As an alternative approach, more comprehensive genotyping techniques that provide additional information like copy number variation in these genes were used to evaluate the diversity of *KIR* genes and their *HLA-C* ligands in humans living in populations with historically varied malaria transmission intensity.

## Methods

### Study samples and populations

Samples from cohorts enrolled at 3 sites in Uganda were utilized, that is, Nagongera Sub-county in Tororo District, a rural area in southeastern Uganda with historically high malaria transmission intensity; Walukuba Sub-county in Jinja District, a peri-urban area near the city of Jinja in south-central Uganda with historically moderate malaria transmission intensity; and Kihihi Sub-county in Kanungu District, a rural area in southwestern Uganda with historically low malaria transmission intensity.

The total population of Nagongera sub-county, Tororo District was 37,500. All participants at this site were recruited within Nagongera Health Center IV, which is the largest public health facility in the sub-county treating an average of 2044 patients per month. Walukuba sub-county, Jinja District has an estimated population of 31,900. Study participants from Walukuba sub-county were recruited within Walukuba health centre IV, the largest public health facility in the sub-county and treating an average of 3198 patients per month. Kihihi is a rural sub-county in Kanungu District with an estimated population of 55,700. Study participants were recruited within Kihihi health centre IV, which is the largest healthcare facility in Kihihi sub-county, a public health facility that treated an average of 1945 patients per month.

To establish these cohorts, all households within the 3 sites were enumerated and mapped, and randomly selected 100 households, that included at least one resident 6 months to 10 years of age, and which were enrolled and followed from August 2011 to September 2013, as previously described [23]. All the participants enrolled in these cohorts provided thick blood smears and a blood sample for genetic analysis. For this study, all participants whose parents consented to future use of their samples were considered. No a priori power calculation was performed.

### Sample collection and DNA purification

Blood samples from previous studies [23] were collected into EDTA tubes, and human genomic DNA was purified from buffy coats using QIAamp DNA Mini Kits (Qiagen), following manufacturer’s instructions using kit inserts with minor modifications. For each sample, 300 μl of buffy coat was mixed with 20 μl of kit protease enzyme solution and then 200 μl of lysis buffer; the mixture was vortexed for 15 s and incubated at 56 °C for 10 min, and then 200 μl of absolute ethanol was added. The mixture was vortexed briefly and transferred to a QIAamp column, and the column was spun for 1 min at 8000 rpm. The column was then washed twice with kit wash buffer, and DNA was eluted by incubating with 80 μl of kit elution buffer at room temperature for 5 min followed by centrifugation at 8000 rpm for 5 min. The DNA concentration was determined using a Qubit fluorimeter (Life Technologies, Carlsbad, CA, USA), and the isolated DNA was stored at − 20 °C.

### Preparation of DNA for multiplex qPCR

To prepare human genomic DNA for *KIR* and *HLA-C* genotyping as well as *KIR* copy number identification, 10 ng samples of genomic DNA (2.5 µl of 4 ng/µl) were aliquoted into 384-well plates using the Hydra 96 micro dispenser (Art Robbins, San Jose, CA, USA) [24]. The DNA was air dried in the plates for subsequent multiplex quantitative PCR assays. Molecular grade water was used in all reactions.

### *KIR* genotyping by high-throughput, multiplex, real-time qPCR

Two pairs of primers were used for each gene, as previously described [25]. Additional *KIR* primers were designed using sequence information from the immuno polymorphism database-*KIR* (IPD-*KIR)* database (release 2.4.0) to detect rare alleles of *KIR2DS5* and *KIR2DL3* (*KIR2DS5, 2DS5rev2*: TCC AGA GGG TCA CTG GGA and *KIR2DL3, 2DL3rev3*: AGA CTC TTG GTC CAT TAC CG) [26]. Samples were genotyped for copy number by multiplex quantitative PCR for all the *KIR* genes (*KIR2DL1, 2DL2, 2DL3, 2DL4, 2DL5, 2DS1, 2DS2, 2DS3, 2DS4, 2DS5, 2DP1, 3DP1, 3DL1, 3DL2, 3DL3, 3DS1*) [24]. Reactions were carried out in quadruplicate to ensure accuracy of the copy number scoring. Two controls with known copy number and one non-template control were included in each run. Two assays for both *3DL1* and *3DL2* genes that target different exons of the genes were included to identify known fusion genes [27], which are carried on a truncated haplotype (with *2DS4* completely deleted) seen in individuals of African descent. There is a drop in copy number for exon 9 of *3DL1* and exon 4 of *3DL2* (i.e., discordance between the exon 4 and exon 9 copy numbers in the same gene) when the fusion gene is present. Assays for *2DS4* variants, *2DS4DEL* (a 22-bp deletion in exon 5 that causes a frameshift mutation) and *2DS4WT* (full-length gene) were also included.

### *HLA-C* genotyping by high-throughput, multiplex, real-time qPCR

A high-throughput real-time qPCR for genotyping *HLA-C* allotypes was developed. For every reaction, KAPA SYBR buffer (5 µl), forward primer (1 µl), reverse primer (1 µl), and water (4 µl) were added to dried DNA in the 384-well plates. *HLA-C1* PCR conditions were: denaturation at 95 °C for 3 min, 5 cycles of 95 °C for 3 s and 72 °C for 30 s, followed by 35 cycles of 95 °C for 3 s and 70 °C for 30 s, dissociation at 60 °C for 1 s, and finally 95 °C. *HLA-C2* PCR conditions were: denaturation at 95 °C for 3 min, 5 cycles of 95 °C for 3 s and 72 °C for 45 s, followed by 40 cycles of 95 °C for 3 s and 70 °C for 45 s, dissociation at 60 °C for 1 s, and finally 95 °C. In each *HLA-C* allotype, primers were used at 5 µM concentrations. Primer combinations for C1 and C2 were: C1 = C1Fa and C1Fb with C1R, and C2 = C2F with C2R, respectively (Table 1). This method was validated against a large range of samples, the HLA Reference Panel from Coriell with known *HLA-C* allotypes, and in families. The sequences for each of the primers used are shown in Table 1.

**Table 1:** *HLA-C* primers for high-throughput qPCR

Primer name	Sequence
C1Fa	GCCGCGAGTCCAAGAGG
C1Fb	GCCGCGAGTCCGAGAGG
C2F	CTGACCGAGTGAACCTGCGGAAA
C2R	GGAGATGGGGAAGGCTCCCCAC
C1R	GCGCAGGTTCCGCAGGC

### KIR and HLA-C genotypes analysis

**Fig. 1:**
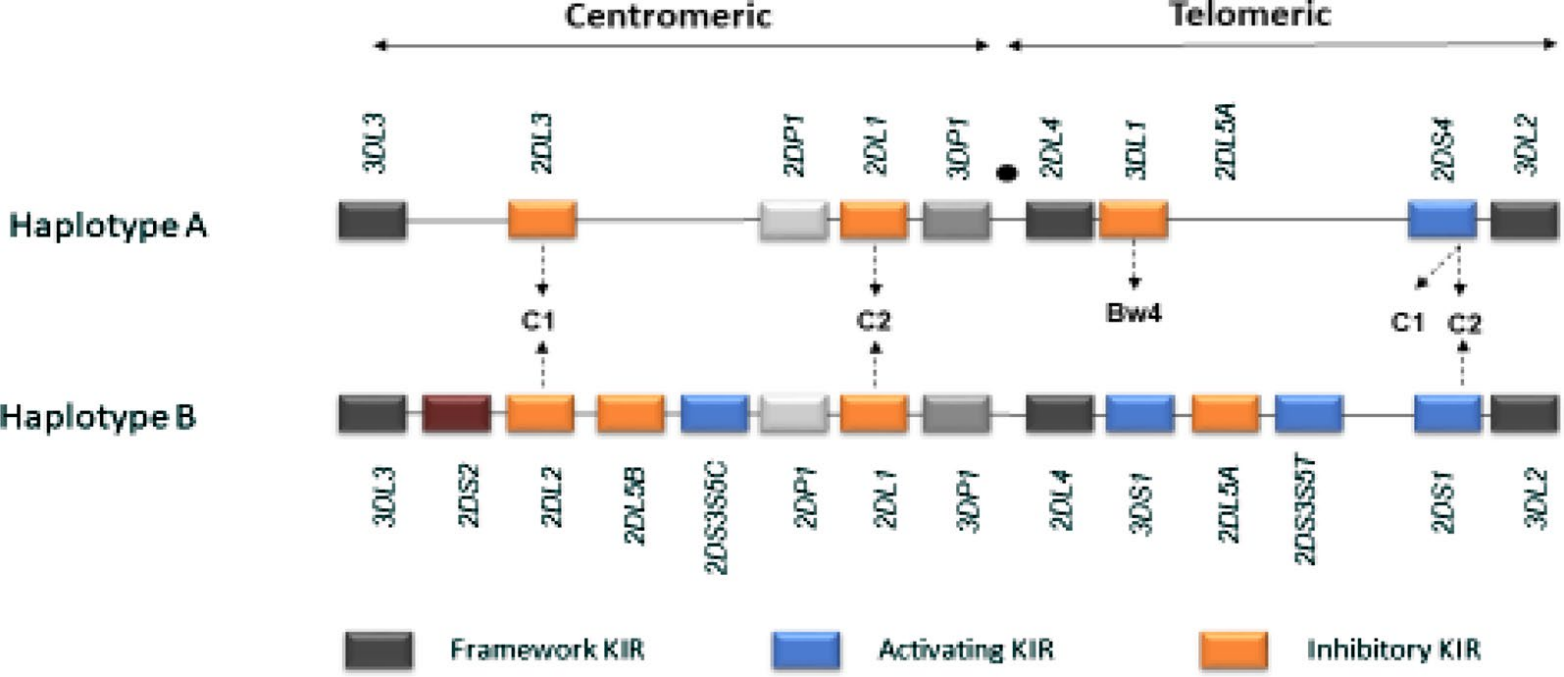
*KIR* haplotypes. KIR A haplotypes (**a**, **b**) are present in all populations worldwide. KIR A haplotype is composed of mainly inhibitory *KIR* except *KIR2DS4*. Allelic polymorphism is very high in the KIR A haplotype (*KIR3DL1*, *3DL2* and *3DL3* exhibit > 100 alleles, and *2DL1* and *2DL3* exhibit ~ 50 alleles). Haplotypes (**b**) has several activating receptors, with variable number of genes and fewer allelic polymorphisms. Some KIR B haplotypes are composed of combinations of haplotypes (**a**, **b**) (CenA-TelB, CenB-TelA). The HLA epitopes bound by some KIRs are known and are indicated as C1, C2 or Bw4

*KIR* genotypes were defined following the recommendations from the 2011 *KIR* workshop that was held at Tammsvik, Stockholm, Sweden [28]. Briefly, the centromeric A region (*cenA)* was defined by the presence of *KIR2DL3* and *KIR2DL1* and absence of any A haplotype gene; the centromeric B (*cenB)* region was defined by presence of any centromeric B haplotype gene (*KIR2DS2* and/or *KIR2DL2*, and/or *2DL5B* and/or centromeric *2DS3/5*). The telomeric A (*telA*) region was defined by *KIR3DL1* and *KIR2DS4* and absence of any A haplotype gene, and the telomeric B (*telB)* region was defined by presence of any centromeric B haplotype gene *(KIR3DS1* and/or *KIR2DS1* and or *2DL5B* and/or telomeric *2DS5*) (Fig. 1). The *KIR* and *HLA-C* genotypes were ascertained according to the Allele Frequency Net Database (http://www.allele.frequencies.net).

### *KIR* Copy number determination by multiplex quantitative PCR

Copy numbers for all *KIR* genes (*KIR2DL1–5, 2DS1–5, 2DS4* (separate assays for the gene, wild-type variant [*2DS4WT*], and deletion variant [*2DS4DEL*]), *2DP1, 3DP1*, *3DL1-3* and *3DS1*) were determined using a Roche Light Cycler 480. Copy numbers were measured by relative quantification analysis of the target *KIR* gene and reference gene (signal transducer and activator of transcription 6; *STAT 6*) using the comparative Cq method [24, 29]. Cq value is the qPCR cycle at which fluorescence from amplification exceeds the background fluorescence (also referred to as threshold cycle, Ct). The ΔΔCq was used to calculate *KIR* copy number. The first ΔCq was calculated by the cycle threshold difference between the target and reference assay of the same sample. The second ΔCq was calculated by the difference of ΔCq values from a test sample and a calibrator sample with known copy number of the target. Two controls with known copy number and one non-template control were included in each run. COPYCALLER software from Applied Biosystems (Foster City, CA, USA) was used to score *KIR* copy numbers. When the Cq of the reference gene was greater than 32 or a data point was more than 4 SD from the mean ΔCq of four replicates, the reaction was not analysed. *KIR* copy number frequencies were calculated for all the samples.

### Statistical methods

Data across the 3 sites was described using frequencies and percentages for categorical variables. Frequencies of *KIR* genes, *KIR* genotypes and *HLA-C* allotypes were calculated by direct counting. Differences in the distribution of *KIR* and *HLA-C* genetic variants within the three populations were compared by Chi-square and Mid-P exact tests. A p-value < 0.05 was considered significant.

## Results

### Characteristics of study participants and populations

Among the 1,344 subjects in the 3 cohorts, 44% were under 5 years of age, and 56% between 5 and 10 years. A recent report defined malaria transmission, prevalence, and incidence in the 3 cohorts (Table 2) [23]. The 3 sites differed markedly, with very high transmission intensity, parasite prevalence and malaria incidence in Tororo District, lower levels of all of these parameters in Kanungu District, and the lowest levels in Jinja District [23, 30]. Of note, malaria transmission was considerably greater in earlier surveys in Jinja District [31], with decreasing transmission likely due to the peri-urban nature of the study area. In Tororo District, transmission has subsequently decreased greatly, after annual indoor residual spraying of insecticides was launched in 2014 [32]. Historically, malaria transmission intensity followed the rank order Tororo > Jinja > Kanungu [31]. The aim of this study was to compare *KIR* and *HLA-C* genetic variants that may have been selected due to differential malaria selection pressures at these sites.

**Table 2:** Characteristics of study participants and populations

	Study sites		
	Tororo	Jinja	Kanungu
Characteristics of sites			
Location	South-eastern	South-central	South-western
Setting	Rural	Peri-urban	Rural
Altitude	695–1443 m	1102–1500 m	886–1329 m
Number of study subjects			
Children below 5 years	340	321	365
Children 5–10 years	106	114	98
Total	446	435	463
Malaria indicators (children)^a^			
Entomological inoculation rate per year	310	2.8	32.0
Parasite prevalence	28.7%	7.4%	9.3%
Malaria incidence per year	2.81	0.43	1.43

^a^Determined August 2011-September 2013 [23]

### Comparative prevalence of *KIR* and *HLA-C* genetic variants at 3 sites in Uganda

Analysis for differential prevalence of *KIR* genes, *KIR* genotypes, *HLA-C* allotypes (*HLA-C1C1, C1C2, C2C2*), centromeric and telomeric *KIR* motifs and *KIR*/*HLA-C* combinations across the 3 populations was carried out (Table 3). More than 90% of samples from all the study populations were successfully analysed for *KIR* and *HLA-C* genetic variants. The prevalence of the inhibitory *KIR* genes *KIR2DL1* and *KIR3DL1* and the activating gene *KIR2DS4* was very high (> 95%). The prevalence of *KIR3DS1* was generally low across the 3 populations, with the lowest prevalence in Kanungu (7.6%) compared to Jinja (18.1%) and Tororo (13.2%). The prevalence of *KIR2DS5* was lower in Kanungu (33.2%) compared to Jinja (43.5%) and Tororo (46.6%). The prevalence of *HLA-C1C2* heterozygotes was higher (53.4%) in all 3 populations compared to homozygous *HLA-C1* (20.1%) or homozygous HLA-C2 (26.5%).

**Table 3:** Distribution of *KIR* and *HLA-C* genetic variants at the 3 sites in Uganda

Genetic variants	Study sites		
	Tororo, N = 438 n (%)	Jinja, N = 414 n (%)	Kanungu, N = 446 n (%)
*KIR* genes			
*2DS2*	224 (51.1)	218 (52.7)	228 (51.1)
*2DL2*	254 (58.0)	240 (58.0)	254 (57.0)
*2DL3*	366 (83.6)	352 (85.0)	380 (85.2)
*2DP1*	432 (98.6)	409 (98.8)	442 (99.1)
*2DL1*	432 (98.6)	409 (98.8)	441 (98.9)
*3DL1*	421 (96.1)	394 (95.2)	439 (98.4)
*3DS1*	58 (13.2)	75 (18.1)	34 (7.6)
*2DL5*	291 (66.4)	264 (63.8)	255 (57.2)
*2DS5*	204 (46.6)	180 (43.5)	148 (33.2)
*2DS3*	92 (21.0)	84 (20.3)	111 (24.9)
*2DS1*	117 (26.7)	126 (30.4)	88 (19.7)
*2DS4*	424 (96.8)	392 (94.7)	438 (98.2)

	Tororo, N = 385 n (%)	Jinja, N = 392 n (%)	Kanungu, N = 433 n (%)
*KIR* genotypes			
*AA*	150 (39.0)	133 (33.9)	171 (39.5)
*BX*	235 (61.0)	259 (66.1)	262 (60.5)

	Tororo, N = 356 n (%)	Jinja, N = 168 n (%)	Kanungu, N = 405 n (%)
*HLA-C* allotypes			
*C1C1*	66 (18.5)	39 (23.2)	75 (18.5)
*C1C2*	195 (54.8)	93 (55.4)	202 (49.9)
*C2C2*	95 (26.7)	36 (21.4)	128 (31.6)

	Tororo, N = 438 n (%)	Jinja, N = 414 n (%)	Kanungu, N = 446 n (%)
Centromeric *KIR* region			
*CenAA*	191 (43.6)	171 (41.3)	190 (42.6)
*CenAB*	175 (40.0)	182 (44.0)	192 (43.1)
*CenBB*	72 (16.4)	61 (14.7)	64 (14.3)

	Tororo, N = 438 n (%)	Jinja, N = 413 n (%)	Kanungu, N = 446 n (%)
Telomeric *KIR* region			
*TelAA*	314 (71.7)	283 (68.5)	356 (79.8)
*TelAB*	115 (26.3)	112 (27.1)	83 (18.6)
*TelBB*	9 (2.0)	18 (4.4)	7 (1.6)

	Tororo, N = 313 n (%)	Jinja, N = 152 n (%)	Kanungu, N = 393 n (%)
Combinations of *KIR* haplotypes/*HLA-C*			
*AA/C1C1*	21 (6.7)	11 (7.3)	22 (5.6)
*AA/C1C2*	62 (19.8)	37 (24.3)	93 (23.7)
*AA/C2C2*	37 (11.8)	11 (7.2)	46 (11.7)
*BX/C1C1*	36 (11.5)	23 (15.1)	50 (12.7)
*BX/C1C2*	109 (34.8)	46 (30.3)	105 (26.7)
*BX/C2C2*	48 (15.4)	24 (15.8)	77 (19.6)

The prevalence of *KIR3DS1, 2DL5, 2DS5*, and *2DS1* genes was significantly lower in Kanungu compared to both Tororo (7.6 vs 13.2%: p = 0.006, 57.2 vs 66.4%: p = 0.005, 33.2 vs 46.6%: p < 0.001, and 19.7% vs 26.7%: p = 0.014, respectively) and Jinja (7.6 vs 18.1%: p < 0.001, 57.2 vs 63.8%: p = 0.048, 33.2 vs 43.5%: p = 0.002 and 19.7 vs 30.4%: p < 0.001, respectively). There was no significant difference in the prevalence of inhibitory *KIR2DL1, 2DL2, 2DL3*, and *3DL1* and activating *KIR2DS2, 2DS3* and *2DS4* (Table 4). The prevalence of homozygous *HLA-C2* was significantly higher in Kanungu (31.6%) compared to Jinja (21.4%), p = 0.043. No significant difference was observed between the prevalence of *HLA-*C2 in Tororo (26.7%) and Kanungu (31.6%), p = 0.296. There was no significant difference in the prevalence of *KIR AA* and *KIR BX* genotypes in Tororo and Jinja (p = 0.145), Tororo and Kanungu (p = 0.877), or Jinja and Kanungu (p = 0.098). Combinations of *KIR* genotypes with *HLA-C* ligands did not differ in the 3 populations. There was no significant difference in the *KIR* centromeric or telomeric motifs across the 3 populations (Table 5).

**Table 4:** Comparative prevalence of *KIR* genes from 3 sites of Uganda with historically varied malaria transmission intensity

Study sites									
Genes	Tororo, N = 438		Jinja, N = 414		Kanungu, N = 446		Mid-P p-values		
	n	%	n	%	n	%	T vs J	T vs K	J vs K
*2DS2*	224	51.1%	218	52.7%	228	51.1%	0.658	0.995	0.652
*2DL2*	254	58.0%	240	58..0%	254	57.0%	0.995	0.754	0.762
*2DL3*	366	83.6%	352	85.0%	380	85.2%	0.558	0.502	0.942
*2DP1*	432	98.6%	409	98.8%	442	99.1%	0.834	0.506	0.654
*2DL1*	432	98.6%	409	98.8%	441	98.9%	0.834	0.739	0.906
*3DL1*	421	96.1%	394	95.2%	439	98.4%	0.497	0.064	0.061
*3DS1*	58	13.2%	75	18.1%	34	7.6%	0.052	*0.006*	* < 0.001*
*2DL5*	291	66.4%	264	63.8%	255	57.2%	0.414	*0.005*	*0.048*
*2DS5*	204	46.6%	180	43.5%	148	33.2%	0.364	* < 0.001*	*0.002*
*2DS3*	92	21.0%	84	20.3%	111	24.9%	0.797	0.170	0.108
*2DS1*	117	26.7%	126	30.4%	88	19.7%	0.229	*0.014*	* < 0.001*
*2DS4*	424	96.8%	392	94.7%	438	98.2%	0.125	0.181	0.059

Mid-P exact p-values for comparisons of *KIR* genes in Tororo *vs* Jinja, Tororo *vs* Kanungu and Jinja *vs* Kanungu

**Table 5:** Comparative prevalence of *KIR* and *HLA-C* genetic variants from 3 sites of Uganda with historically varied malaria transmission intensity

Study sites									
	Tororo, N = 385		Jinja, N = 392		Kanungu, N = 433		p-values		
	n	(%)	n	(%)	n	(%)	T vs J	T vs K	J vs K
*KIR* genotypes									
* AA*	150	39.0%	133	33.9%	171	39.5%	0.145	0.877	0.098
* BX*	235	61.0%	259	66.1%	262	60.5%			

	Tororo, N = 356		Jinja, N = 168		Kanungu, N = 405		p-values		
	n	(%)	n	(%)	n	(%)	T vs J	T vs K	J vs K
*HLA-C*									
* C1C1*	66	18.5%	39	23.2%	75	18.5%	0.285	0.296	0.043
* C1C2*	195	54.8%	93	55.4%	202	49.9%			
* C2C2*	95	26.7%	36	21.4%	128	31.6%			

	Tororo, N = 120		Jinja, N = 59		Kanungu, N = 161		p-values		
	n	(%)	n	(%)	n	(%)	T vs J	T vs K	J vs K
*KIRAA/HLA-C*									
* AA/C1C1*	21	17.5%	11	18.6%	22	13.7%	0.213	0.537	0.282
* AA/C1C2*	62	51.7%	37	62.8%	93	57.8%			
* AA/C2C2*	37	30.8%	11	18.6%	46	28.5%			

	Tororo, N = 193		Jinja, N = 93		Kanungu, N = 232		p-values		
	n	(%)	n	(%)	n	(%)	T vs J	T vs K	J vs K
*KIRBX/HLA-C*									
* BX/C1C1*	36	18.6%	23	24.7%	50	21.5%	0.424	0.062	0.424
* BX/C1C2*	109	56.5%	46	49.5%	105	45.3%			
* BX/C2C2*	48	24.9%	24	25.8%	77	33.2%			

	Tororo, N = 438		Jinja, N = 414		Kanungu, N = 446		p-values		
	n	(%)	n	(%)	n	(%)	T vs J	T vs K	J vs K
Centromeric *KIR* motif									
* CenAA*	191	43.6%	171	41.3%	190	42.6%	0.478	0.552	0.928
* CenAB*	175	40.0%	182	44.0%	192	43.0%			
* CenBB*	72	16.4%	61	14.7%	64	14.4%			

	Tororo, N = 438		Jinja, N = 413		Kanungu, N = 446		p-values		
	n	(%)	n	(%)	n	(%)	T vs J	T vs K	J vs K
Telomeric *KIR* Motif									
* TelAA*	314	71.7%	283	68.5%	326	73.1%	0.141	0.088	0.061
* TelAB*	115	26.3%	112	27.1%	113	25.3%			
* TelBB*	9	2.0%	18	4.4%	7	1.6%			

p-values for comparisons of the prevalence of KIR AA vs BX genotypes, HLA (C1C1) vs C1C2 vs C2C2, KIR/HLA (AA/C1C1) vs AA/C1C2 vs AA/C2C2, KIR/HLA (BX/C1C1) vs BX/C1C2 vs BX/C2C2, centromeric (CenAA) vs CenAB vs CenBB, telomeric (TelAA) vs TelAB vs TelBB in Tororo (T), Jinja (J) and Kanungu (K) districts were determined using Fisher’s exact test.

### Copy number variation in *KIR* genes and malaria transmission intensity

Determination of whether CNV in *KIR* genes is influenced by malaria transmission intensity was done by comparing *KIR* CNV in children from the 3 populations. Comparisons were done for inhibitory *KIR2DL1, 2DL2, 2DL3*, and *2DL5*, and the activating *KIR2DS2* and *2DS5*. All the *KIR* genes, including framework genes, were subject to CNV. The majority of study participants (over 90%) had 0–2 copies. However, there was no significant difference in *KIR* CNV across the 3 study populations (Table 6), suggesting that CNV in *KIR* genes may not be influenced by *P. falciparum* pathogen pressure.

**Table 6:** Comparison of CNV in *KIR* genes in populations with historically varied malaria transmission intensity

	Tororo		Jinja		Kanungu		p-value
	N = 438	F (%)	N = 414	F (%)	N = 446	F (%)	
*KIR2DL1* CNV							
0	6	1.4	5	1.2	5	1.2	0.985
1	104	23.7	99	23.9	101	22.6	
2	328	74.9	310	74.9	340	76.2	
*KIR2DL2* CNV							
0	184	42	174	42	192	43.1	0.834
1	220	50.2	199	48.1	216	48.4	
2	34	7.8	41	9.9	38	8.5	
*KIR2DL3* CNV							
0	72	16.4	62	15	66	14.8	0.195
1	154	35.2	171	41.3	191	42.8	
2	212	48.4	181	43.7	189	42.4	
*KIR2DS2* CNV							
0	214	48.8	196	47.3	218	48.9	0.387
1	207	47.3	189	45.7	204	45.7	
2	17	3.9	29	7	24	5.4	
*KIR2DS5* CNV							
0	336	76.7	324	78.3	372	83.4	0.144
1	98	22.3	86	20.7	71	16	
2	4	1	4	1	3	0.6	
*KIR2DL5* CNV							
0	292	66.8	282	68.2	318	71.4	0.650
1	138	31.4	126	30.5	121	27	
2	8	1.8	6	1.3	7	1.6	

CNV is copy number variation of *KIR* genes. The value can be 0, 1 or 2 in these populations, F is the frequency of participants with the different copies of *KIR* genes

## Discussion

Most studies about genetic variation in KIR and HLA class I molecules and malaria have focused mainly on protection from severe malaria [33]. The aim of this study was to determine whether *KIR* and *HLA-C* genetic variants and CNV in *KIR* genes from 3 populations of Uganda with historically varied malaria transmission intensity have been shaped by selection pressure from falciparum malaria. Appreciation of malaria transmission prior to recent intensive control efforts and urbanization suggests a rank order for historical transmission intensity of Tororo > Jinja > Kanungu [31]. Thus, the measured prevalence of *KIR* genes and their *HLA*-*C* ligands in populations with historically varied malaria transmission was aimed at understanding impacts of malaria evolutionary pressure on *KIR* and *HLA* genes.

There was high *KIR* diversity in the 3 studied populations, as has been seen in previous studies in Uganda [20] and in other African populations [34]. Generally, the frequency of *KIR3DS1* was low across the 3 populations, similar to that reported in previous studies from other African populations [35]. The frequency of *KIR3DS1* was significantly lower in Kanungu compared to Tororo and Jinja, implying that *KIR3DS1* could have been positively selected for in Tororo and Jinja to offer some advantage against malaria. The prevalence of *KIR2DS5* and *KIR2DL5* genes was significantly lower in Kanungu. Interestingly, results from a previous study in Nigeria demonstrated that *KIR2DS5* and *KIR2DL5* genes were associated with reduced parasitaemia [36]. The *KIR3DS1*, *KIR2DL5*, *KIR2DS5* and *KIR2DS1* genes can be present together on a particular haplotype in sub-Saharan Africans [37]. Differences in the prevalence of this haplotype across the three sites could potentially be explained by the selective pressure imposed by malaria. If so, the responsible gene or genes on the haplotype are not known, but *KIR3DS1* has a low frequency and is present on few other haplotypes in Ugandans [38]. This gene is more prevalent in other populations, including Europeans [39], suggesting that it is selected against in Uganda or it evolved outside Africa [35]. The observed differences may be due, in part, to genetic differences between the ethnic groups principally inhabiting these populations. Indeed, in the previous study from these cohorts, it was observed that the populations of Tororo and Kanungu were homogeneous, based on language groups, but the Jinja population had ethnic groups from all over Uganda [40]. Although the specific ligands and expression details for *KIR2DS3* and *KIR2DS5* are yet to be defined, it is speculated that under functionally relevant combinations these activating genes, in conjunction with their putative ligands, may increase the threshold of NK cell activation and subsequent recruitment of other immune factors that mediate protection against malaria.

Although there was no significant difference in *KIR/HLA-C* combinations across the three sites in Uganda, it should be noted that, interactions between *KIR* and their *HLA-C* ligands within an individual play a key role in modulating the activity of NK cells [41]. For instance, the presence of particular *HLA-C* allotypes and inhibitory *KIR2DL1, KIR2DL2* and *KIR2DL3* genes determines the strength of NK cell inhibition during malaria infection [33]. The best characterized *KIR-HLA* ligand interactions are *KIR2DL1* with the *HLA-C2* subgroup and *KIR2DL2/L3* with the *HLA-C1* subgroup. Generally, *KIR2DL1/HLA-C2* provides the strongest inhibition, followed by *KIR2DL2/HLAC1*, and *KIR2DL3/HLA-C1* [42, 43]. *HLA-C1/C1* individuals are only able to receive inhibitory signals via *KIR2DL2* and *KIR2DL3*, whereas *HLA-C2/C2* individuals receive inhibitory signals predominantly via *KIR2DL1*, and heterozygous individuals have the ligand for all three of these *KIR* genes [44]. Lower *KIR* inhibition may allow unrestrained NK cell activation that could contribute to immune-mediated pathology. This would be consistent with the theory that mechanisms that prevent malaria infection and those that prevent severe disease are distinct and may have a balancing effect on the maintenance of different *KIR* and their *HLA* ligands in malaria-endemic populations.

The role of *KIR/HLA* compound genotypes during falciparum malaria requires more attention given that malaria parasites spend most of the life cycle outside of HLA-expressing cells. Sporozoites infect hepatocytes after injection by mosquitoes. This is the only stage in the parasite replicative life cycle which is within an HLA-expressing host cell (39). Because erythrocyte membranes contain little to no HLA (42), it is postulated that the influence of KIR on cell-mediated anti-parasite immunity may occur primarily during the liver stage. This implies that cellular immune responses play an important role in restricting *P. falciparum* infection. During the blood stage, KIR-expressing effector cells may respond more strongly to an HLA-devoid cell due to the loss of inhibitory signalling via inhibitory KIR (43). KIR inhibition may also influence the clearance of parasites through antibody-dependent cellular cytotoxity (44, 45).

Previous studies have indicated that variation in *KIR* copy number, which leads to expression differences [14], may be important for susceptibility to some diseases. For example, CNV of *KIR3DL1/S1* influences HIV control [45] and expression differences of *KIR2DL3*, interacting with *HLA-C*, may have a profound effect on resolution of hepatitis C virus infection [46]. However, there was no significant difference between *KIR* CNV across the three populations of Uganda.

Although different *KIR* and *HLA* variants may have been selected in different populations primarily due to differential risk of malaria, the role of other infectious pathogens that are prevalent in these malaria-endemic populations should not be overlooked, as they may also have exerted selective pressure on the evolution of *KIR* and *HLA*. Therefore, the role of other co-infections should be considered in future studies involving *KIR* and malaria, especially in populations affected by many infectious pathogens.

This study had some limitations. First, the genotyping technique for both *KIR* and *HLA-C* could not give detailed information up to the allele level. Second, other *HLA* class I genes, for instance *HLA-B* (e.g., *HLA Bw4* and *HLA Bw6* allotypes), which may play a role in malaria risk were not looked at. Nevertheless, analysis for *HLA-C* allotypes, which are the major ligands for *KIR* genes, was done. Third, the status of *KIR* genes and their *HLA-C* ligands remain unknown in a larger part of Uganda that was not covered in this study given its limited coverage. Despite these limitations, description of the genetic diversity of *KIR* and their *HLA-C* ligands in populations with historically varied malaria transmission intensity offered an opportunity to identify *KIR* and *HLA-C* genetic variants that are under positive selection and are potentially important in protection against malaria.

## Conclusions

This study has provided baseline information about differences in the prevalence *KIR* genes and their *HLA-C* ligands in populations of Uganda with historically varied malaria transmission intensity. The *KIR3DS1*, *KIR2DL5*, *KIR2DS5*, and *KIR2DS1* genes may partly explain differences in transmission intensity of malaria since these genes have been positively selected for in places with historically high malaria transmission intensity. This is the largest cohort ever studied investigating *KIR*, *HLA-C* and malaria risk. A new high-throughput real-time PCR assay for HLA-C genotyping has been developed from this study. This will be useful in disease association studies that involve larger cohorts.

## Data Availability

The datasets utilized for this study are available from the corresponding author on reasonable request.

## References

[1] WHO (2019). World Malaria Report 2019.

[2] Weatherall DJ (2008). Genetic variation and susceptibility to infection: the red cell and malaria. Br J Haematol.

[3] Kwiatkowski DP (2005). How malaria has affected the human genome and what human genetics can teach us about malaria. Am J Hum Genet.

[4] Hedrick PW (2011). Population genetics of malaria resistance in humans. Heredity.

[5] Carter R, Mendis KN (2002). Evolutionary and historical aspects of the burden of malaria. Clin Microbiol Rev.

[6] López C, Saravia C, Gómez Camacho A, Hoebeke J, Patarroyo M (2010). Mechanisms of genetically-based resistance to malaria. Gene.

[7] Manjurano A, Clark TG, Nadjm B, Mtove G, Wangai H, Sepulveda N (2012). Candidate human genetic polymorphisms and severe malaria in a Tanzanian population. PLoS ONE.

[8] Toure O, Konate S, Sissoko S, Niangaly A, Barry A, Sall AH (2012). Candidate polymorphisms and severe malaria in a Malian population. PLoS ONE.

[9] Band G, Le QS, Clarke GM, Kivinen K, Hubbart C, Jeffreys AE (2019). Insights into malaria susceptibility using genome-wide data on 17,000 individuals from Africa Asia and Oceania. Nat Commun.

[10] Burrack KS, Hart GT, Hamilton SE (2019). Contributions of natural killer cells to the immune response against Plasmodium. Malar J.

[11] Wolf A-S, Sherratt S, Riley EM (2017). NK Cells: uncertain allies against malaria. Front Immunol.

[12] Khakoo S, Carrington M (2006). KIR and disease: a model system or system of models?. Immunol Rev.

[13] Hellmann I, Lim SY, Gelman RS, Letvin NL (2011). Association of activating KIR copy number variation of NK cells with containment of SIV replication in rhesus monkeys. PLoS Pathog.

[14] Béziat V, Traherne JA, Liu LL, Jayaraman J, Enqvist M, Larsson S (2013). Influence of KIR gene copy number on natural killer cell education. Blood.

[15] Doolan DL, Dobaño C, Baird JK (2009). Acquired immunity to malaria. Clin Microbiol Rev.

[16] Tukwasibwe S, Nakimuli A, Traherne J, Chazara O, Jayaraman J, Trowsdale J (2020). Variations in killer-cell immunoglobulin-like receptor and human leukocyte antigen genes and immunity to malaria. Cell Mol Immunol.

[17] Kulkarni S, Martin MP, Carrington M (2008). The Yin and Yang of HLA and KIR in human disease. Semin Immunol.

[18] Radwan J, Babik W, Kaufman J, Lenz TL, Winternitz J (2020). Advances in the Evolutionary Understanding of MHC Polymorphism. Trends Genet.

[19] Meyer D, Aguiar CVR, Bitarello BD, Brandt CDY, Nunes K (2018). A genomic perspective on HLA evolution. Immunogenetics.

[20] Nakimuli A, Chazara O, Farrell L, Hiby SE, Tukwasibwe S, Knee O (2013). Killer cell immunoglobulin-like receptor (KIR) genes and their HLA-C ligands in a Ugandan population. Immunogenetics.

[21] Chazara O, Xiong S, Moffett A (2011). Maternal KIR and fetal HLA-C: a fine balance. J Leukoc Biol.

[22] Parham P (2005). MHC class I molecules and KIRs in human history, health and survival. Nat Rev Immunol.

[23] Kamya MR, Arinaitwe E, Wanzira H, Katureebe A, Barusya C, Kigozi SP (2015). Malaria transmission, infection, and disease at three sites with varied transmission intensity in Uganda: implications for malaria control. Am J Trop Med Hyg.

[24] Jiang W, Johnson C, Simecek N, López-Álvarez MR, Di D, Trowsdale J (2016). qKAT: a high-throughput qPCR method for KIR gene copy number and haplotype determination. Genome Med.

[25] Hiby SE, Walker JJ, O'Shaughnessy KM, Redman CW, Carrington M, Trowsdale J (2004). Combinations of maternal KIR and fetal HLA-C genes influence the risk of preeclampsia and reproductive success. J Exp Med.

[26] Robinson JT, Thorvaldsdóttir H, Winckler W, Guttman M, Lander ES, Getz G (2011). Integrative genomics viewer. Nat Biotechnol.

[27] Norman PJ, Abi-Rached L, Gendzekhadze K, Hammond JA, Moesta AK, Sharma D (2009). Meiotic recombination generates rich diversity in NK cell receptor genes, alleles, and haplotypes. Genome Res.

[28] Malmberg K-J, Michaëlsson J, Parham P, Ljunggren H-G (2011). Killer cell immunoglobulin-like receptor workshop: Insights into evolution, genetics, function, and translation. Immunity.

[29] Jayaraman J, Kirgizova V, Di D, Johnson C, Jiang W, Traherne JA (2019). KAT: quantitative semi-automated typing of killer-cell immunoglobulin-like receptor genes. J Vis Exp..

[30] Rek J, Katrak S, Obasi H, Nayebare P, Katureebe A, Kakande E (2016). Characterizing microscopic and submicroscopic malaria parasitaemia at three sites with varied transmission intensity in Uganda. Malar J.

[31] Yeka A, Gasasira A, Mpimbaza A, Achan J, Nankabirwa J, Nsobya S (2012). Malaria in Uganda: challenges to control on the long road to elimination: I. Epidemiology and current control efforts. Acta Trop..

[32] Katureebe A, Zinszer K, Arinaitwe E, Rek J, Kakande E, Charland K (2016). Measures of malaria burden after long-lasting insecticidal net distribution and indoor residual spraying at three sites in Uganda: a prospective observational study. PLoS Med.

[33] Hirayasu K, Ohashi J, Kashiwase K, Hananantachai H, Naka I, Ogawa A (2012). Significant association of KIR2DL3-HLA-C1 combination with cerebral malaria and implications for co-evolution of KIR and HLA. PLoS Pathog.

[34] Norman PJ, Hollenbach JA, Nemat-Gorgani N, Guethlein LA, Hilton HG, Pando MJ (2013). Co-evolution of human leukocyte antigen (HLA) class I ligands with killer-cell immunoglobulin-like receptors (KIR) in a genetically diverse population of sub-Saharan Africans. PLoS Genet.

[35] Norman PJ, Abi-Rached L, Gendzekhadze K, Korbel D, Gleimer M, Rowley D (2007). Unusual selection on the KIR3DL1/S1 natural killer cell receptor in Africans. Nat Genet.

[36] Ademola S, Amodu O, Yindom L-M, Conway D, Aka P, Bakare A (2014). Killer-cell immunoglobulin-like receptors and falciparum malaria in southwest Nigeria. Hum Immunol.

[37] Nemat-Gorgani N, Guethlein LA (2019). Diversity of KIR, HLA Class I, and their interactions in seven populations of sub-Saharan Africans. J Immunol.

[38] Nakimuli A, Chazara O, Hiby SE, Farrell L, Tukwasibwe S, Jayaraman J (2015). A KIR B centromeric region present in Africans but not Europeans protects pregnant women from pre-eclampsia. Proc Natl Acad Sci USA.

[39] Körner C, Altfeld M (2012). Role of KIR3DS1 in human diseases. Front Immunol.

[40] Walakira A, Tukwasibwe S, Kiggundu M, Verra F, Kakeeto P, Ruhamyankaka E (2017). Marked variation in prevalence of malaria-protective human genetic polymorphisms across Uganda. Infect Genet Evol.

[41] Pende D, Falco M, Vitale M, Cantoni C, Vitale C, Munari E (2019). Killer Ig-like receptors (KIRs): their role in nk cell modulation and developments leading to their clinical exploitation. Front Immunol.

[42] Moesta AK, Norman PJ, Yawata M, Yawata N, Gleimer M, Parham P (2008). Synergistic polymorphism at two positions distal to the ligand-binding site makes KIR2DL2 a stronger receptor for HLA-C than KIR2DL3. J Immunol.

[43] Carrington M, Wang S, Martin MP, Gao X, Schiffman M, Cheng J (2005). Hierarchy of resistance to cervical neoplasia mediated by combinations of killer immunoglobulin-like receptor and human leukocyte antigen loci. J Exp Med.

[44] Biassoni R, Falco M, Cambiaggi A, Costa P, Verdiani S, Pende D (1995). Amino acid substitutions can influence the natural killer (NK)-mediated recognition of HLA-C molecules. Role of serine-77 and lysine-80 in the target cell protection from lysis mediated by "group 2" or "group 1" NK clones. J Exp Med..

[45] Pelak K, Need AC, Fellay J, Shianna KV, Feng S, Urban TJ (2011). Copy number variation of KIR genes influences HIV-1 control. PLoS Biol.

[46] Khakoo SI, Thio CL, Martin MP, Brooks CR, Gao X, Astemborski J (2004). HLA and NK cell inhibitory receptor genes in resolving hepatitis C virus infection. Science.

